# Increased competency of registered dietitian nutritionists in physical examination skills after simulation-based education in the United States

**DOI:** 10.3352/jeehp.2020.17.40

**Published:** 2020-12-14

**Authors:** Elizabeth MacQuillan, Jennifer Ford, Kristin Baird

**Affiliations:** Department of Allied Health Sciences, College of Health Professions, Grand Valley State University, Allendale, MI, USA; Hallym University, Korea

**Keywords:** Clinical competence, Motivation, Nutritionists, Physical examination, United States

## Abstract

**Purpose:**

This study aimed to translate simulation-based dietitian nutritionist education to clinical competency attainment in a group of practicing registered dietitian nutritionists (RDNs). Using a standardized instrument to measure performance on a newly-required clinical skill, the nutrition-focused physical exam (NFPE), competence was measured both before and after a simulation-based education (SBE) session.

**Methods:**

Eighteen practicing RDNs were recruited by their employer, Spectrum Health. Following a pre-briefing session, participants completed an initial 10-minute encounter, performing NFPE on a standardized patient (SP). Next, participants completed a 90-minute SBE training session on skills within the NFPE, including hands-on practice and role play, followed by a post-training SP encounter. Video recordings of the SP encounters were scored to assess competence in 7 skill areas within the NFPE. Scores were analyzed for participants’ initial competence and change in competence.

**Results:**

The proportions of participants with initial competence ranged from 0% to 44% across the 7 skill areas assessed. The only competency where participants initially scored in the “meets expectations” range was “approach to the patient.” When raw competence scores were assessed for changes from pre- to post-SBE training, the paired t-test indicated significant increases in all 7 competency areas following the simulation-based training (P<0.001).

**Conclusion:**

This study showed the effectiveness of a SBE training program for increasing competence scores of practicing RDNs on a defined clinical skill.

## Introduction

### Background/rationale

Simulation-based education (SBE) in professional training programs has expanded in recent years [1,2]. The foundational components of SBE that set it apart from other pedagogical techniques are the inclusion of pre- and de-briefing discussions between facilitators and learners, establishment of a safe space for learners to practice and make mistakes, and the inclusion of realistic patient case scenarios in the learning environment [1,2]. Despite the more widespread inclusion of SBE in both educational and clinical learning environments, little research has established guidelines for how to quantify the effects of SBE in terms of changes in clinical competence of learners or improved patient care and safety [1-4]. Assessment of clinical skills competence is also a current need for dietetics educators, due to the recent development of a competency-based future education model of dietetics education [5]. An example of a clinical skill recently made mandatory for all registered dietitian nutritionists (RDNs) and graduates of dietetics education programs to demonstrate is the nutrition-focused physical exam (NFPE) [6].

The NFPE has been established as a key tool of dietetics practitioners for the identification of malnutrition and micronutrient imbalances in adult and pediatric patients [7]. As of 2017, the Accreditation Council on Education in Nutrition and Dietetics has required accredited dietetics programs to provide instruction in the NFPE [6]. However, recent studies of practicing RDNs showed that many RDNs reported feeling unprepared to include the NFPE in their own clinical practice [8]. As a result, health care systems that employ RDNs are seeking training opportunities to increase utilization of this valuable clinical skill [8,9]. As a physical examination requiring physical contact with patients, a training utilizing the simulated environment and actual persons portraying patient cases (standardized patients, SPs) is an ideal pedagogical method for teaching and practice of the NFPE skill.

### Objectives

This study examined a group of practicing RDNs to identify changes in competence scores for clinical skills after participating in a 90-minute SBE session. Further, this study obtained a snapshot of existing competence levels of practicing RDNs for the NFPE, a required clinical skill. The research questions were as follows: first, what is the current rate of competence in the NFPE skill among practicing clinical RDNs?; second, are years of clinical experience, frequency of performing NFPE, and the education level of RDNs related with their initial competence scores in the NFPE?; third, does simulation-based training increase practicing clinicians’ competence in specific skills (e.g., NFPE)?; and fourth, what variables (experience, frequency of performing NFPE, and education level) are associated with the greatest gains in competence from before to after simulation-based training?

## Methods

### Ethics statement

Ethical approval was obtained from the Grand Valley State University Human Subjects Institutional Review Board as exempt research (#18-220-H).

### Study design

This pre-and post-intervention comparison study included RDNs employed at Spectrum Health. This article was described according to the STROBE (Strengthening the Reporting of Observational Studies in Epidemiology) statement, available from: https://www.strobe-statement.org/.

### Setting

This study took place in a university simulation center setting, specifically the SP suite. This suite consisted of outpatient exam rooms outfitted with a video camera system that enabled the recording of patient examinations conducted in a realistic environment. Specifically, the SPs were adults employed by the simulation center and trained to portray a variety of simulated patient cases. Training and data collection occurred in 2 groups, held in January and February 2020. The same researchers conducted the SBE training and scored the video-recorded SP encounters for both groups.

### Participants

The RDNs were selected for training by their employer, as part of a professional development activity offering, and selection was not based on level of experience, area of practice, or level of interest. A total of 18 RDNs participated in the study in the 2 groups (containing 10 and 8 participants, respectively).

### Variables

Initially, all RDN participants were asked to complete a survey on their level of education, time since dietetic registration, and frequency of performing NFPE in the course of their typical practice. The remaining variables analyzed in this study were performance scores on each of the 7 competencies that make up the overall NFPE skill.

### Data sources/measurement

#### Pre-interventional test

The competency scores were awarded by a team of 3 researchers, among whom use of the standardized assessment tool had previously been tested for satisfactory inter-rater reliability. The pre-training data were obtained from an assessment of video-recorded SP encounters conducted prior to the 90-minute SBE training. Initially, participants were led in a 10-minute pre-briefing activity where SP case information was shared. As part of the pre-briefing, participants were informed that the simulation center is a safe space for practice and development of skills, a component of the pre-briefing that has been discussed by other researchers as a key component of SBE [1,2]. Following the pre-briefing, participants were asked to conduct a pre-training 10-minute NFPE encounter with an assigned SP. A paper documentation form containing all of the 7 competency areas on the assessment tool was provided to each participant prior to each SP encounter.

#### Educational intervention

The SBE education methods utilized in the 90-minute training provided to participants included: pre-briefing and de-briefing, role playing, and practicing skills on both SPs and peers. This session was taught and facilitated by the 3 researchers, all faculty members from the graduate-level dietetics program at the sponsoring university. The training included an evidence-based presentation of skills required for competence in NFPE, as determined by guidelines established by the Academy of Nutrition and Dietetics [10,11]. Traditional instruction was enhanced by demonstrations of the NFPE skill by the faculty, followed by time for hands-on practice of skills by the participants on their peers utilizing role-playing with patient information. The equipment utilized for the training and practice included stethoscopes for the abdominal exam and dynamometers for measurements of functional status.

#### Post-interventional test

After completing the training, the participants performed a second 10-minute NFPE encounter with a different SP. Following the second encounter, all participants met back at the classroom for a 20-minute de-briefing session to discuss what went well, what was difficult, and the connections between NFPE findings and the given case information for each SP.

#### Measurement

NFPE skills were assessed by reviewing video recordings of the SP encounters. Recordings were made accessible to the researchers through an encrypted website for the purpose of assigning a competence score. The instrument used to assess competence from the video encounters was developed based on evidence-based practice guidelines for clinical competence assessment and using the guidelines of the Academy of Nutrition and Dietetics for Nutrition-Focused Physical Exam [10,11]. The tool is comprised of 7 competencies (1: approach to patient; 2: micronutrient deficiency/excess; 3: head/face; 4: upper body; 5: lower body; 6: abdominal exam; 7: functional status assessment) broken into performance indicators with criteria statements to aid raters in scoring performance on a 9-point scale. The instrument, developed by these authors, has demonstrated excellent content validity, inter-rater reliability, and internal consistency in standardization testing. For content validity, a total of 7 expert dietetics educators and clinical managers rated the NFPE competency assessment tool on a 4-point relevance scale. The item content validity index scores calculated for each of the 7 competencies assessed by the tool ranged from 0.86 to 1.00, indicating excellent relevance of items on the scoring tool. When ratings on all 7 competencies, at both the pre- and post-training time points, were assessed by 3 raters viewing the same recordings, the overall Fleiss kappa results based on a total of 140 observations indicated extremely strong agreement (κ=0.863; 95% confidence interval [CI], 0.860–0.866; P<0.001). When scores for each competency were considered individually and assessed for agreement between the 3 raters, the Fleiss kappa results again confirmed excellent agreement, with competency-specific scores ranged from κ=0.726 for competency 7 to κ=1.00 for competency 6. The relatedness of scores among the 7 competencies were analyzed by calculating the inter-class correlation coefficient (ICC) for the competencies (ICC, 0.913; 95% CI, 0.879–0.940; P<0.001). Additionally, the Cronbach alpha statistic was calculated for the 7 competencies, with the result of α=0.913, indicating excellent internal consistency between items. The competency scoring instrument can be viewed in Supplement 1.

### Bias

To reduce the potential for confirmation bias in scoring by the researchers, the raters were blinded to whether the participant videos they scored were from the pre- or post-training time points. The 2 patient cases portrayed and the skills performed in both the pre- and post-training encounters were identical; each participant saw one case pre-training and the other case post-training, but the same 2 cases were used in both sessions. Additionally, videos were marked with a code number (not names) for participants, and researchers did not have access to the code list linking data from the survey of experience level and area of practice until after all videos were scored.

### Study size

A total of 18 RDNs participated in this study. Each RDN completed both a pre- and a post-training SP encounter in which they performed the NFPE skill. The G*Power tool (Heinrich-Heine-Universität Düsseldorf, Düsseldorf, Germany; http://www.gpower.hhu.de/) was used to estimate the sample size for the paired t-test. The a priori G*Power test indicated that the required sample size was 16 based on a 2-tailed test, an effect size of 0.9, alpha error of 0.05, and a power of 0.9. The actual power was 0.915.

### Statistical methods

Variables such as time in practice, highest educational degree earned, and frequency of using NFPE in practice were considered in the analyses in order to detect any effects on competence. Statistical analyses conducted in IBM SPSS ver. 25.0 (IBM Corp., Armonk, NY, USA) included: first, descriptive statistics of the subject population; second, the percent of RDNs competent at the initial time point; third, the change in competence scores following remediation training, with mean competence scores compared using the paired t-test; and fourth, analysis of variance (ANOVA) to determine whether the analyzed variables (length of time in practice, education level, and area of practice) were associated with significant differences in mean competence scores from the initial assessment to the post-training re-assessment. For analyses, scores were considered both on the 9-point scale and categorically, in 3 levels. Categories were considered to better indicate the clinically relevant groups of “below expectations” (scores of 1–3), “meets expectations” (scores of 4–6), and “exceeds expectations” (scores of 7–9). For a binary analysis of initial competence, scores within the category of “meets” or “exceeds” expectations were considered as “competent.” These categorical outcome analyses were included in order to identify differences in NFPE skills that would be relevant and meaningful in the professional health care setting.

## Results

### Participants

The subjects included 18 RDNs employed at Spectrum Health, a private health system in the Midwest region of the United States. All 18 participants attended the 90-minute training and completed both pre- and post-training NFPE assessments. The raw data file is available in Dataset 1 and 2.

### Descriptive data

The mean number of years in practice as an RDN for the participants was 9.6 years (standard deviation=7.7). Most participants (n=13, 72.2%) had earned a bachelor’s degree as their highest earned degree. The largest category reported for the average number of times performing NFPE in a week was 15 or more times per week (n=8, 44.4%), followed by 8–14 times (n=4, 22.2%). The complete results of the survey are shown in Table 1.

### Main results

#### Competence at the initial time point

When competence as a binary variable was assessed for each item, the percentage of RDNs competent at the initial time-point ranged from 0% (competency 6, abdominal exam) to 44% (competency 1, approach to the patient). The only competency where participants scored in the “meets expectations” range initially was competency 1 (approach to the patient; mean score=3.94 out of a maximum score of 9; standard deviation=1.18). The initial rates and scores for each competency are shown in Table 2.

#### Relationships of years of clinical experience, frequency performing NFPE, and education level of RDNs with their initial competence scores

One-way ANOVA of the initial competence rates by years in practice, frequency of performing NFPE, and highest degree completed detected no significant differences in any of the competency areas.

#### Change in competence scores following SBE training

When competence scores on the 9-point scale were assessed for changes from pre- to post-SBE training, significant increases in competence scores were observed in all 7 competency areas. The greatest increase in competence scores among the RDN participants occurred in competency 6 (abdominal exam), with a mean increase of 3.72 points (95% CI, 3.12–4.26), followed by competency 7 (functional status assessment; mean increase, 3.31; 95% CI, 2.69–3.93). The smallest increase from before to after training occurred in competency 1 (approach to the patient; mean increase, 1.27; 95% CI, 0.73–1.81). Further investigation into the significance of differences between mean scores at the pre-training and post-training time points using the paired t-test revealed significant increases in all 7 competency areas following the simulation-based training (P<0.001). In terms of the binary distinction between “meets expectations” and “below expectations,” across all 7 competencies, on average 15 out of the 18 participants (83.3%) scored as competent following the SBE training. Complete results of the competency score change and the paired t-test are shown in Table 3.

#### Contributions of variables to changes in competence following SBE

Relationship between changes in competence level and variables—years of experience as an RDN, number of times per week performing NFPE, and highest degree completed—were examined using 1-way ANOVA. No significant relationships were found between the change in mean score from pre- to post-training and any groupings by experience and education.

## Discussion

### Key results

Based on the competence change results (Table 3), it appears that simulation-based training was an effective method for teaching these skills, and participants achieved a mean score following the training that was within the range of competence. The simulation-based training provided in this study produced significant increases in all 7 competencies and, for the 6 competencies where participants scored “below expectations” prior to training (competencies 2–7), the post-training scores fell within the range of competence (scores between 4 and 6, equivalent to “meets expectations”).

### Limitations

Limitations of this study include the fact that the researchers who developed the competency assessment tool also rated participants at the pre-/post-training time points. Although steps were taken to reduce confirmation bias, such as blinding researchers to whether videos were from a pre- or post-training time point, it is possible that subconscious rating inflation could have occurred due to the investment of the raters in the study.

### Interpretation

The competencies with the lowest initial scores (competency 6, abdominal exam and competency 7, functional status assessment) were the same competencies with the greatest increase in scores after the simulation-based training. These areas are advanced-level skills that are not always included in basic instruction on performance of the NFPE [12,13]. This may be because they require specialized equipment (a stethoscope for abdominal auscultation and a dynamometer for hand grip strength measurement) not traditionally provided for the use of RDNs in clinical settings [12,13]. Therefore, the simulation-based training may have been the first time many participants were exposed to these skills.

Although the largest group of participants (n=8, 44.4%) indicated on the experience survey that they performed NFPE on average 15 or more times per week, the competence scores were initially low across all 7 skill areas. This may have originated from the simulation lab setting, which was new to all of the participants, or from participants’ unfamiliarity with some of the instrumentation provided for use (for example, the stethoscope for abdominal exam and dynamometer for functional status/grip strength).

### Generalizability

The results of this study serve multiple purposes, for different stakeholders. The results will serve clinical managers and educators in the field of dietetics by providing data on competence levels of practicing RDNs in the NFPE skill. Additionally, the findings will serve to move the field of health professions education forward by translating SBE to competence gains of practicing clinicians, as opposed to assessments of learner-perceived traits, as previous studies have done [3,14].

### Conclusion

This study demonstrates a method of quantifying the changes in clinical skill competence that occurred as a result of SBE training. Within the field of dietetics, other clinical skills that could be tested using SBE include feeding tube placement, such as guided nasogastric tube placement at the bedside. Future work should seek to explore potential differences in the effectiveness of SBE at competency-building in clinicians with different levels of experience and education, as well as in health professions students.

## Figures and Tables

**Table 1.** Demographic and professional characteristics of the participants (n=18)

Characteristic	No. of participants(%)
Years as RDN	
1–4	8 (44.4)
5–9	2 (11.1)
10–19	5 (27.8)
≥20	3 (16.7)
Highest degree earned	
Bachelor’s	13 (72.2)
Master’s	5 (27.8)
Average no. of times in a week that the respondent performed NFPE	
0–2	3 (16.7)
3–7	3 (16.7)
8–14	4 (22.2)
≥15	8 (44.4)

RDN, registered dietitian nutritionist; NFPE, nutrition-focused physical exam.

**Table 2.** Registered dietitians’ initial competence scores on the nutrition-focused physical exam (n=18)

Competency	No. of participants (%) scoring as competent at initial assessment	Mean±SD score at initial assessment^a)^
1: Approach to patient	8 (44.4)	3.94±1.18
2: Micronutrient deficiency/excess	3 (16.7)	2.52±1.05
3: Head/face	6 (33.3)	3.17±0.94
4: Upper body	4 (22.2)	3.00±0.75
5: Lower body	3 (16.7)	2.53±0.098
6: Abdominal exam	0	1.11±0.47
7: Functional status assessment	2 (11.1)	1.81±1.26

SD, standard deviation.

a) Competence scores based on a 9-point scale, where 1–3=below expectations, 4–6=meets expectations, and 7–9=exceeds expectations.

**Table 3.** Change in mean competence scores on the nutrition-focused physical exam from before to after simulation-based training, based on paired t-test analysis

Competence area	Mean±SD score at post-test^a)^	Mean difference (post-test–pre-test score)	t-value (degrees of freedom)	P-value
1: Approach to patient	5.21±0.82	1.27 (0.73–1.81)	4.94 (17)	0.001
2: Micronutrient deficiency/excess	4.52±1.03	2.00 (1.41–2.49)	8.64 (17)	<0.001
3: Head/face	4.97±0.95	1.81 (1.10–2.51)	5.41 (17)	<0.001
4: Upper body	4.75±1.13	1.75 (1.13–2.37)	5.99 (17)	<0.001
5: Lower body	4.00±1.55	1.47 (0.79–2.15)	4.61 (17)	<0.001
6: Abdominal exam	4.83±0.99	3.72 (3.19–4.26)	14.7 (17)	<0.001
7: Functional status assessment	5.11±0.82	3.31 (2.69–3.93)	11.2 (17)	<0.001

SD, standard deviation.

a) Competence scores based on a 9-point scale, where 1–3=below expectations, 4–6=meets expectations, and 7–9=exceeds expectations.

## Footnotes

**Data availability** Data files are available from Harvard Dataverse: https://doi.org/10.7910/DVN/U5QSNJ Dataset 1. NFPE competence raw scores. Dataset 2. Competence change from pre- to post-training.

## Supplementary materials

Supplementary files are available from Harvard Dataverse: https://doi.org/10.7910/DVN/U5QSNJ

Supplement 1. Grand Valley State University Nutrition-Focused Physical Exam Competency Tool.

Supplement 2. Audio recording of the abstract.

## References

[1] Morse C, Fey M, Kardong-Edgren S, Mullen A, Barlow M, Barwick S (2019). The changing landscape of simulation-based
education. Am J Nurs.

[2] Bogossian F, Cooper S, Kelly M, Levett-Jones T, McKenna L, Slark J, Seaton P (2018). Best practice in clinical simulation education: are we there
yet?: a cross-sectional survey of simulation in Australian and New Zealand
pre-registration nursing education. Collegian.

[3] McGaghie WC, Issenberg SB, Cohen ER, Barsuk JH, Wayne DB (2012). Translational educational research: a necessity for effective
health-care improvement. Chest.

[4] Posner GD, Clark ML, Grant VJ (2017). Simulation in the clinical setting: towards a standard
lexicon. Adv Simul (Lond).

[5] Accreditation Council for Education in Nutrition and
Dietetics (2019). Future education model standards for accredited graduate programs in
dietetics.

[6] Accreditation Council for Education in Nutrition and
Dietetics (2016). Crosswalk of knowledge and competency statements for CP, DI, DPD, FDE,
IDE programs.

[7] White JV, Guenter P, Jensen G, Malone A, Schofield M, Academy Malnutrition Work Group, A.S.P.E.N. Malnutrition Task Force, A.S.P.E.N. Board of Directors (2012). Consensus statement: Academy of Nutrition and Dietetics and
American Society for Parenteral and Enteral Nutrition: characteristics
recommended for the identification and documentation of adult malnutrition
(undernutrition). JPEN J Parenter Enteral Nutr.

[8] Griswold K, Rogers D, Sauer KL, Kellogg Leibovitz P, Finn JR (2016). Entry-level dietetics practice today: results from the 2015
commission on dietetic registration entry-level dietetics practice
audit. J Acad Nutr Diet.

[9] Esper DH, Pohle-Krauza RJ, Leson SM (2010). Nutrition focused physical assessment: preceptors’
education, application and perceived barriers. J Am Diet Assoc.

[10] Kidd JL, Radler DR, Brody RA, Parrott JS (2012). Nutrition focused physical assessment (NFPA) teaching practices
in accreditation council for education in nutrition and dietetics
(ACEND)-accredited dietetic internships and coordinated programs
(DIs/CPs). J Acad Nutr Diet.

[11] Mordarski B (2016). Nutrition-focused physical exam hands-on training
workshop. J Acad Nutr Diet.

[12] Mordarski BA, Hand RK (2019). Patterns in adult malnutrition assessment and diagnosis by
registered dietitian nutritionists: 2014-2017. J Acad Nutr Diet.

[13] Moulder C (2018). Nutrition-focused physical exam training by a peer champion
increases use of and perceived confidence and comfort in physical
exam. J Acad Nutr Diet.

[14] Issenberg SB, Ringsted C, Ostergaard D, Dieckmann P (2011). Setting a research agenda for simulation-based healthcare
education: a synthesis of the outcome from an Utstein style
meeting. Simul Healthc.

