# Supplementary material for: Increased competency of registered dietitian nutritionists in physical examination skills after simulation-based education in the United States
Source: J Educ Eval Health Prof. 2020 Dec 14;17:40. doi: 10.3352/jeehp.2020.17.40 (PMC7857069; doi:10.3352/jeehp.2020.17.40)
Supplement: Supplementary file 3 — Supplement 1. Grand Valley State University Nutrition-Focused Physical Exam Competency Tool. [file jeehp-17-40-suppl1.pdf]

Figure 1.

GVSU Nutrition-Focused Physical Exam Competency Tool

Instructions: Review the Competencies, Performance Indicators and Criteria below. The criteria represent the standards scorers should look for when assigning a score for each performance indicator. Scores will be aggregated by competency and scored by performance indicator.

Competency	Performance Indicator	Criteria statement
**1**	**Approach to Patient**	
1.1	Greeting	Introduced self to patient/family (1st name only, title, and/or department)
1.2	Description of NFPE and overall purpose of exam	Provided rationale for examination request, asked patient permission to exam, requested patient identifiers (DOB, first name, last name)
1.3	Hand Hygiene	Before entering and exiting patient room: washed in, washed out with antibacterial soap and water for $\geq$ 15 seconds or used alcohol based sanitizer; before physical examination: wash/dry hands thoroughly before donning gloves and after taking gloves off
1.4	Talked through transitions of exam & findings	Provided explanation prior/during examination of each area as appropriate; ask clarifying questions as needed to confirm findings
1.5	Friendly conclusion to encounter	Verbally stated an appreciation phrase to patient (i.e. It was nice meeting you, thank you for allowing me to complete a NFPE). Return room to patient-preferred state (ex: bed position, lighting, TV, etc.)
2	**Micronutrient Deficiency/Excess**	

Figure 1.

2.1	Skin assessment	Temperature, Texture (dry scaly), Color (pallor, jaundice),Turgor, Wounds / rashes / lesions, bruising
2.2	Hair assessment	Assess scalp – shape and symmetry, masses. dry, dandruff, lesions / sores / psoriasis, Assess hair distribution pattern / quantity (alopecia), color variations, corkscrew hair, dullness
2.3	Nails assessment	Color, length, texture, configuration /shape / symmetry (angle contour), lesions, palpate the nail
2.4	Eyes assessment	Night blindness, dryness, Bitot's spots (Vitamin A)<br>Itching, burning, corneal inflammation (Riboflavin and niacin)<br>Pale conjunctiva (Iron, folate, B12)<br>Scleral icterus (carotene, bilirubin)
2.5	Oral cavity assessment (mouth, lips, gum, tongue, teeth)	Angular stomatitis/cheilosis (B-complex vitamins)<br>Angular stomatitis/cheilosis (B-complex vitamins)<br>Glossitis, magenta/red beefy tongue (Riboflavin, niacin, folate, B12, iron, protein)<br>Bleeding gums, poor dentition (Vitamin C)<br>Dysgeusia (Zinc)
**3**	**Head/Face**	
3.1	Fat assessment	Orbital region (orbital fat pads)
3.2	Muscle assessment	Temple (temporalis)

Figure 1.

**4**	**Upper Body**	
4.1	Fat assessment	• Upper arm (triceps)<br>• Mid-axillary (just above iliac crest)<br>• Ribs
4.2	Muscle assessment	•Clavicle (pectoralis major, deltoid)<br>•Shoulder (deltoid)<br>•Scapula (trapezius)<br>•Hand (interosseous)
**5**	**Lower Body**	
5.1	Muscle assessment	• Thigh/knee (quadriceps, femoris)<br>• Calf (gastrocnemius)
5.2	Fluid Accumulation observed and documented	Assess feet / ankles, sacrum for edema, dehydration (skin turgor/tenting). Note type of edema, location, and severity (+1 pitting edema, etc.)<br><br>Note and document presence of ascites or anasarca.
**6**	**Abdominal Exam**	
6.1	Explain purpose of exam	Explain importance and purpose of exam to patient before beginning.
6.2	Auscultation for bowel sounds	Listen in all four quadrants, note any findings, ask follow-up questions of patient as needed.
**7**	**Functional Status Assessment**	
7.1	Grip strength measurement explained (purpose & procedure)	Explain procedure. Adjust hand position of dynamometer to correct distance. Confirm dynamometer handle is in correct position. Place dynamometer in hand of patient with strap around patient's wrist, gently support base.

Figure 1.

		Patient’s arm at 90-degree angle, held at side. Arm not resting on chair arm. Sitting position. If able, feet flat on ground, shoulder width apart. Patient is instructed to grip (3 – 5 seconds) with 30-60 second rest between measurements on one hand. Inform patient when to begin and when to stop.
7.2	Three measurements taken and recorded	3 measurements on the right / 3 measurements on the left<br>Clinician reads and documents results, along with documenting hand position of dynamometer (i.e. in 2nd position, right hand)

Figure 1.

**Initial or Reassessment (circle):**	**Name:**

**Comp No.**	**Criteria/Performance Indicator**	**Below expectations**			**Met expectations**			**Exceeded expectations**			**N/O**	**Rationale for Scoring**
**1**	**Approach to Patient**											
1.1	Greeting	1	2	3	4	5	6	7	8	9		
1.2	Description of NFPE and overall purpose of exam	1	2	3	4	5	6	7	8	9		
1.3	Cleaned Hands	1	2	3	4	5	6	7	8	9		
1.4	Talked through transitions of exam & findings	1	2	3	4	5	6	7	8	9		
1.5	Friendly conclusion to encounter	1	2	3	4	5	6	7	8	9		
**2**	**Micronutrient Deficiency / Excess**											
2.1	Skin assessment	1	2	3	4	5	6	7	8	9		
2.2	Hair assessment	1	2	3	4	5	6	7	8	9		
2.3	Nails assessment	1	2	3	4	5	6	7	8	9		
2.4	Eyes assessment	1	2	3	4	5	6	7	8	9		
2.5	Oral cavity assessment	1	2	3	4	5	6	7	8	9		
**3**	**Head/Face**											
3.1	Fat assessment	1	2	3	4	5	6	7	8	9		
3.2	Muscle assessment	1	2	3	4	5	6	7	8	9		
**4**	**Upper Body**											
4.1	Fat assessment	1	2	3	4	5	6	7	8	9		
4.2	Muscle assessment	1	2	3	4	5	6	7	8	9		
**5**	**Lower Body**											
5.1	Muscle assessment	1	2	3	4	5	6	7	8	9		
5.2	Fluid status assessed	1	2	3	4	5	6	7	8	9		
**6**	**Abdominal Exam**											
6.1	Explain purpose of exam	1	2	3	4	5	6	7	8	9		
6.2	Auscultation in all four quadrants	1	2	3	4	5	6	7	8	9		
**7**	**Functional Status Assessment**											
7.1	Grip strength measurement explained (purpose & procedure)	1	2	3	4	5	6	7	8	9		
7.2	Three measurements taken and recorded	1	2	3	4	5	6	7	8	9		
